# Testing the Feasibility and Preliminary Efficacy of an 8-Week Exercise and Compensatory Eating Intervention

**DOI:** 10.3390/nu10070923

**Published:** 2018-07-19

**Authors:** Jessica S. West, Kym J. Guelfi, James A. Dimmock, Ben Jackson

**Affiliations:** School of Human Sciences, The University of Western Australia, 35 Stirling Highway, Crawley, Perth, WA 6009, Australia; kym.guelfi@uwa.edu.au (K.J.G.); james.dimmock@uwa.edu.au (J.A.D.); ben.jackson@uwa.edu.au (B.J.)

**Keywords:** compensatory snacking, justification, nutrition, physical activity

## Abstract

The aim of this study was to evaluate the feasibility and preliminary efficacy of an intervention comprised of regular exercise alongside educational and motivational support for participants’ avoidance of unhealthy compensatory eating. Forty-five sedentary individuals were randomized to an 8-week exercise plus compensatory eating avoidance program (CEAP; *n* = 24), or an 8-week exercise intervention only (control; *n* = 21). The feasibility and preliminary efficacy of the intervention were assessed using quantitative measures and supplemented with written responses to open-ended questions. The CEAP workshop was well-received; however, self-reported use of some of the included behavior change strategies was lower than expected. Post-intervention, there was evidence of reduced self-reported compensatory eating for participants in the CEAP group but not controls, with CEAP participants also reporting greater use of coping plans relative to controls post-intervention. The exercise program had benefits for waist circumference, body fat percentage, blood pressure, and cardiovascular fitness; however, improvements were similar between groups. Taken together, the results of this study indicate that the CEAP is feasible and may reduce compensatory eating around exercise; however, this effect is small. Potential modifications to the CEAP are discussed within the paper.

## 1. Introduction

Exercise is commonly employed as a means of optimizing health and managing weight. However, not all individuals attain their desired long-term goals (e.g., weight loss) through exercise interventions [1,2,3]. One reason why exercise interventions may fail to provide desired outcomes for some individuals is due to compensatory eating—the consumption of unhealthy, energy-dense snack foods and drinks following, or in advance of, planned exercise [4,5,6]. Although individuals may be aware that these types of foods and drinks are counter-productive to their long-term health and wellbeing goals, they may still consciously reward themselves for exercising (or in anticipation of exercising) through deliberate and reflective justifications (e.g., “I deserve a piece of chocolate cake because I just had a good workout”). This act of “licensing” may be considered a type of compensatory health belief (CHB); the belief that the negative effects of one behavior (e.g., consuming a piece of chocolate cake) can be reversed or “neutralized" by what is considered a positive behavior (e.g., exercise) [7]. Although from the CHB literature, one might draw conclusions about the temporal ordering of the paired unhealthy (first) and healthy (second/subsequent) behaviors [7,8], at a conceptual level, it is entirely possible that one might “compensate” (or as we have termed it, “license”) in the opposite direction, whereby individuals first engage in a healthy behavior (e.g., exercise) and subsequently—as a result of that healthy behavior—reward, license, or allow themselves to indulge in an unhealthy behavior (e.g., a pleasurable, high-fat snack). On this basis, and consistent with the previous work by West et al., [9], we use the term “licensing” to reflect a specific type of CHB (i.e., a specific temporal ordering in which a healthy behavior proceeds an unhealthy behavior) in the present manuscript.

Exercise and food/drink are a common CHB pairing, with the majority of participants in a recent study reporting compensatory eating following exercise [10]. Additional studies have also shown that unhealthy snack foods and drinks are commonly used as rewards for engaging in exercise [6,9]. Although exercise and compensatory eating are commonly paired [10], no research has been undertaken to explore whether, or how much, the beliefs [9] or behaviors [10] about unhealthy compensatory eating around exercise are modifiable. In addition, research is needed to reveal the extent to which programs aimed at reducing compensatory eating may influence dietary behavior during—and the health outcomes stemming from—exercise interventions. In terms of potential methods suited to addressing these gaps, the provision of CHB information in the form of a targeted workshop related to compensatory eating (in response to, or anticipation of, exercise) might be an effective supplement to an exercise program. Such an approach may offer a valuable opportunity to provide exercisers with: (1) information about the frequency and negative consequences of compensatory eating, and (2) encouragement and behavior change techniques designed to help them avoid compensatory eating around exercise. As justification cues (e.g., exercise) may influence behavior outside of conscious awareness [11,12], providing information about the behavior–health link, along with other behavioral change strategies such as creating an awareness of one’s behaviors through self-monitoring [13], may maximize the effectiveness of a behavior change intervention [14]. Barriers to long-term health and wellbeing (e.g., daily habits, time, lack of willpower, etc.) may also be present, even when an individual is aware of the negative effects of poor health choices [15]. Thus, taking an active approach to self-regulating health behaviors may act as a bridge between healthy intentions and behaviors, and help address potential barriers to behavior change [13,16]. In relation to the provision of behavior change strategies, it is noteworthy that although a full taxonomy of behavior change techniques is available for interventions focused on exercise and healthy eating [17], some specific techniques may be more beneficial than others [14,18]. For instance, the use of self-monitoring appears to be particularly important in these types of interventions [13,18]. A vast amount of research has also shown techniques including goal setting, action and coping planning, using reminders and prompts, and enlisting social support, are beneficial for health behavior change [18,19,20,21]. With this in mind, we sought to develop a program to assist individuals to avoid compensatory eating around exercise by integrating each of these techniques.

At present, the feasibility and potential efficacy of complementing an exercise program with information provision and behavior change techniques targeting the avoidance of unhealthy compensatory eating is unknown. A recognized and crucial first stage in the development and optimization of any intervention is to assess elements associated with the feasibility of the program [22]. Effect sizes, acceptability of testing procedures, participants’ satisfaction with the program, and rates of recruitment and retention are all important components of a feasibility study that provide insight into the structure, effectiveness, and potential improvement of an intervention [23]. The primary purpose of this study, therefore, was to test the feasibility and preliminary efficacy of an intervention in which participants were provided (or not provided) with education and behavior change content regarding the avoidance of compensatory eating surrounding an 8-week supervised exercise program. Feasibility was assessed by documenting recruitment, adherence, and attrition rates, together with obtaining objective and subjective quantitative data alongside written feedback to open-ended questions regarding the intervention and its components. The preliminary efficacy of the intervention for altering CHBs was assessed by comparing the intervention group with an exercise-only control group. With the purpose of providing effect size information for a suite of relevant objective outcomes (and those that may be of interest in interventions of this kind in the future), we also assessed relative change for both groups of participants on body composition, blood pressure, fasting blood glucose, and cardiovascular fitness. 

## 2. Materials and Methods 

### 2.1. Participant Recruitment 

Participants were recruited through newsletter advertisements and emails within the authors’ institution, various community clubs, and local businesses. The program was referred to as a “healthy lifestyle intervention” in all recruitment materials, and prospective participants were informed that the purpose of the study was to understand the effects of regular exercise on psychological and physiological markers of health and wellbeing. Participants were aged 18–45 years with a body mass index (BMI) between 18.5 and 29.99 kg/m^2^ and were eligible for the study if they did not meet the American College of Sports Medicine (ACSM) exercise recommendations of 150 min of moderate intensity exercise per week [24]. Exclusion criteria were any pre-existing conditions that caused pain or prohibited exercise, a diagnosed eating disorder, or pregnancy. Further details of participants at baseline are presented in Table 1. All participants provided written informed consent and the procedures were approved by the Human Research Ethics Committee at the authors’ institution. 

### 2.2. Study Design 

**Figure 1:**
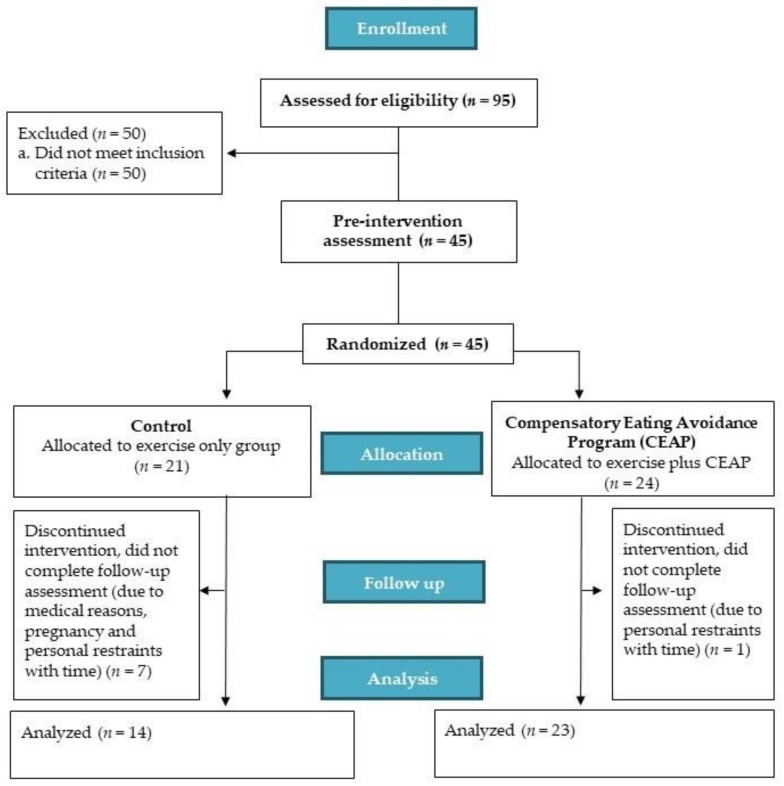
Flow diagram of the progress between the exercise only and exercise plus CEAP intervention groups through the stages.

This study was designed as a randomized between-group feasibility trial (see Figure 1 for flow diagram) with all testing conducted within the authors’ institution. After an initial screening call, eligible participants attended a laboratory for baseline (pre-intervention) assessments (detailed below). After baseline assessment, each participant was randomized to: (1) an 8-week exercise intervention (control), or (2) an 8-week exercise intervention including a CEAP. Randomization was conducted by members of the research team external to the lead author (who was blinded so as to avoid bias during her delivery of exercise sessions and completion of testing assessments). The lead author, therefore, remained blinded as to participants’ treatment allocation until participants had completed all intervention and assessment procedures. The CEAP required participants to attend a 1.5 h workshop within the first two weeks of commencing their 8-week supervised exercise training. These CEAP participants were asked to provide feedback about the workshop through closed- and open-ended questions immediately after the workshop and at the end of the intervention. Following the 8-week intervention period, each participant again attended the laboratory for post-intervention assessments, after which they were debriefed about the true aims of the study. Participants assigned to the exercise only (control) condition received the option of attending the CEAP workshop after data collection for the study was completed. 

### 2.3. Exercise Intervention 

All participants completed the same 8-week supervised exercise program. Briefly, this program involved each participant attending the laboratory three times per week and exercising at self-selected times for 8 weeks. All sessions were supervised, with up to 10 people exercising at a given time. Despite the group-based nature of the exercise, interaction between participants was minimized by inviting individuals to focus on their own regime and by encouraging them to bring their own music, books, or videos to the exercise sessions. Attendance at these sessions was noted, along with the details of each session (duration, ratings of perceived exertion (RPE)) to determine compliance. Additional physical activity outside of the supervised intervention was also recorded at the end of each exercise session.

All participants started with 30 min of moderate intensity exercise on a cycle ergometer in the first week of the intervention, targeting a heart rate of 65–70% of age-predicted maximum heart rate (HR_max_; monitored by a HR monitor (Polar Electro Oy, Kempele, Finland)) and an RPE between 12 and 14 [25], reflecting a realistic exercise duration and frequency goal for previously sedentary participants [26]. Cycling session duration increased incrementally to 35 min in the second week, 40 min in the third and fourth weeks, 45 min in the fifth and sixth weeks, and 50 min for the final 2 weeks of the intervention to ensure the ACSM-recommended 150 min moderate intensity exercise per week was being reached by the final stages of the program. The target heart rate for these later sessions was 65–75% HR_max_, with the RPE maintained at 12–14. 

### 2.4. Compensatory Eating Avoidance Program 

The CEAP workshop was 1.5 h in duration and covered three major modules. The first was an introductory module outlining the benefits of, and general recommendations for, exercise and healthy nutrition [27]. The second module covered education about compensatory health beliefs and behaviors in which participants were informed about (a) the prevalence of compensatory eating [10], (b) the potential negative effects of compensatory beliefs/behaviors [4,7,28], and (c) a reflection activity, in which participants were invited to discuss their own beliefs/behaviors around exercise and compensatory eating. The third was a final behavior change module in which participants (a) set goals to avoid compensatory eating based on the Specific, Measureable, Assignable (replaced with Achievable), Realistic and Time-related (S.M.A.R.T) framework [29], (b) created action and coping plans regarding these goals and their avoidance of compensatory eating [20] around their exercise participation, and (c) were encouraged to implement cues and reminders, engage in self-monitoring, and enlist meaningful social support to avoid compensatory eating [17]. Considering the positive effects that self-monitoring has demonstrated in previous behavior change interventions [13], we provided a self-monitoring food diary to participants in the CEAP condition to encourage their use of these strategies. Following the workshop, and for the remainder of the exercise period, the program also included the delivery of weekly reminder emails that targeted different components of the workshop, such as encouraging participants to: (1) reflect on their progress, (2) maintain their self-monitoring, (3) consider necessary revisions to their health and wellbeing goals, (4) monitor and revise, where necessary, their action and coping plans, (5) reflect on their use of prompts and reminders, and (6) initiate conversations with family or friends about their health and wellbeing goals. 

### 2.5. Assessment of Feasbility of the Exercise Intervention 

To assess the feasibility and acceptability of the intervention, all aspects of recruitment were documented, as were adherence rates and attrition in the two groups. The duration and intensity of supervised exercise was documented, together with any additional exercise sessions completed outside of the supervised program. Finally, to assess any group differences in perceived enjoyment of the program as a whole, the interest/enjoyment subscale of the Intrinsic Motivation Inventory (IMI) [30] was completed by both groups post intervention.

#### 2.5.1. CEAP Workshop

To assess the dose received during the CEAP workshop, participants in the CEAP provided feedback on their perceptions of the workshop immediately after the workshop in the form of a seven-item survey that measured perceptions about the usefulness and comprehensibility of workshop material (items listed in Table 2 in the results). All items were scored on a 5-point Likert scale ranging from 1 (strongly disagree) to 5 (strongly agree), with higher scores denoting more positive evaluations.

#### 2.5.2. Use of Behavior Change Techniques

Following the completion of the intervention, a questionnaire was administered to assess the degree to which participants in the CEAP: (1) followed through with their goals (e.g., “During the 8-week intervention, to what extent did you stick to the goals you specified in the Healthy Lifestyle Workshop”), and (2) used the specified behavior change strategies during the intervention (e.g., “During the 8-week intervention, to what extent did you use the self-monitoring diary to monitor your food and drink intake around exercise”). All statements used the common stem “During the 8-week intervention, to what extent did you…” and were scored on a 5-point Likert scale ranging from 1 (not at all) to 5 (very much), with higher scores indicating greater adherence to goals/strategies. All five items are presented in Table 2 in the results section.

An additional six-item survey was administered in the post-intervention assessment to evaluate the degree to which participants (in both conditions) had made action and coping plans around healthy eating behaviors and exercise [31]. The common stem “During the healthy lifestyle intervention, I made detailed plans regarding...” was used for all items, with three action planning items targeting when, what, and how often to eat and drink, and the coping planning items assessing plans for addressing barriers (e.g., “...what to do in difficult situations in order to optimize healthy eating and drinking behaviors around exercise”). All items were assessed on a 4-point Likert scale ranging from 1 (completely disagree) to 4 (completely agree). Separate mean scores were calculated for action and coping planning, with higher scores indicating greater use of plans. Internal consistency for scores derived from the action planning (α = 0.69) and coping planning (α = 0.84) instruments was acceptable. 

#### 2.5.3. Open-Ended Feedback and Recommendations 

At the end of the program, participants in the CEAP were provided with seven open-ended questions. Two items were designed to assess the perceived effect of the CEAP on compensatory eating beliefs (i.e., “To what extent did the workshop encourage you to change your beliefs about compensatory/licensing eating and drinking behavior (around exercise)? Please explain why or why not”) and actual behaviors (i.e., “did you feel that your eating and drinking behavior around exercise was modified as a result of the workshop? Please explain how and why, or why not”). Four items assessed how and why each of the behavior change techniques (i.e., goal setting and planning, enlisting social support, self-monitoring, and prompts) had or had not been implemented during the program. Participants were asked, for example, to “explain how you used the goal setting and planning strategies to enhance your health and wellbeing goals. If you didn’t use them much, please elaborate on why you didn’t, and tell us how we could have supported you to use this tool more”. In the seventh and final question, participants were asked to provide broad comments and recommendations regarding their thoughts about how to optimize and refine the CEAP for future use. 

### 2.6. Assessment of Preliminanary Efficacy of the Intervention 

Preliminary efficacy of the intervention was assessed by measuring several relevant variables pre- and post-intervention. Participants attended the laboratory in a fasted state between 06:00 and 10:00, having refrained from exercise for 24 h prior to both the pre- and post-intervention assessments. The specific time of attendance was selected by the participant at the pre-intervention assessment and was repeated for the post-intervention assessment. Upon arrival to the laboratory, participants completed a number of questionnaires to assess background variables (age, gender, socio-economic rating, highest form of education, ethnicity, and day of menstrual cycle for women), exercise behavior (Godin Leisure Time Exercise Questionnaire) [32], and cognitive restraint of eating (Three Factor Eating Questionnaire: TFEQ-R18) [33].

#### 2.6.1. Compensatory Beliefs and Behaviors

The primary outcome, CHBs regarding unhealthy food/drinks and exercise, was assessed at the completion of the physical testing in accordance with previous research [9]. Using the common stem, “After engaging in exercise…”, participants completed six items on a 7-point scale ranging from 1 (strongly disagree) to 7 (strongly agree). An example item includes “I think I can have unhealthy snacks because I’ve earned them”. An average CHB score was calculated, with higher scores reflecting greater endorsement of unhealthy food/drink licensing around exercise. Consistent with previous studies [9], scores derived from the scale displayed evidence of acceptable internal consistency both pre- (α = 0.84) and post-intervention (α = 0.80). In addition, the first question of the Compensatory Eating Motives Questionnaire (CEMQ) [10] was administered to examine whether participants believed that they engaged in compensatory behaviors prior to or following exercise (“Do you eat more on days that you exercise”); this item was scored on a Likert scale anchored at 1 (never/almost never) to 4 (almost always/always). Only the first question of the scale was used given that the scale subsequently excludes participants if they do not select 2 (labelled “sometimes”) or above on the initial question, and because the West et al. [9] instrument was considered to be more appropriate for our study aim (i.e., beliefs about unhealthy food/drinks around exercise). 

#### 2.6.2. Health and Fitness

Secondary outcomes included the assessment of resting heart rate, blood pressure, body composition, and fasting blood glucose, which were measured at the beginning of the pre- and post-intervention laboratory testing sessions after sitting quietly for 10 min while filling in the initial questionnaires listed above. Body composition, including fat and lean muscle mass percentages, was assessed using dual energy X-ray absorptiometry (DEXA) (GE Lunar iDXA Advance from General Electrical Healthcare, Madison, WI, USA). Fasting glucose was determined from a capillary blood sample obtained from the fingertip in a microcuvette and analyzed using a HemoCue Glucose 201+ System (HemoCue AB, Ängelholm, Sweden). Participants then completed a modified version of the Aerobic Power Index test [34] on a cycle ergometer (Exertech Ex-10 front access cycle ergometer (Repco Cycle Company, Huntingdale, Victoria, Australia)) to assess cardiovascular fitness. Briefly, this procedure involved exercise of progressively increasing intensity until a heart rate equivalent to 80% of age-predicted maximum was achieved, with fitness expressed as power output (W) at 80% of HR_max_ [35].

#### 2.6.3. Snack Intake

Upon completion of the physical testing, participants were left alone in the laboratory for 15 min to complete the final questionnaires including the CHB scale and CEMQ, in addition to a measure of exercise motivation (Behavioral Regulation in Exercise Questionnaire: BREQ3) [36,37]. During this time participants had access to an assortment of pre-weighed snack foods. Five food items were selected based upon palatability and variety in sensory characteristics and health status (both unhealthy and healthy options) including: Maltesers (Mars Chocolate Australia, Ballarat, Australia) chocolate chip cookies (Woolworths homebrand, Bella Vista, Australia), confectionary sweet jelly snakes (Woolworths homebrand, Bella Vista, Australia), bananas, apples, and water. To minimize the influence of environmental factors on eating behavior, the same food was presented in the same position on a tray for all participants at both the pre- and post-intervention assessments. All items were re-weighed after the participant had left the laboratory to allow for calculation of total energy intake for each item and category (i.e., unhealthy: maltesers, chocolate chip cookies, confectionary; healthy: apples, bananas) based on the manufacturers nutrition label, or a nutrition software package (FoodWorks v 4.2.0; Xyris Software, Brisbane, Australia). 

### 2.7. Data Analysis 

When participants failed to respond to a whole variable or subscale, data were not replaced. Data within the amotivation subscale of the BREQ3 were missing completely at random (Little’s [38] Chi-square test was non-significant, *χ*^2^ (3) 0.243, *p* = 0.970). All other subscales of the BREQ3 had no missing data. Missing data for the BREQ3 amotivation subscale were replaced using the expectation maximization procedure [39]. No other missing data were observed for measures that could be replaced (i.e., compensatory health beliefs, cognitive restraint of eating, action and coping planning and ratings of enjoyment of the intervention). Eight participants did not complete the intervention and were excluded from analysis, and one participant in the CEAP was excluded from any food-related assessments (i.e., cognitive restraint of eating, compensatory health beliefs and behaviors, action and coping planning and energy intake), due to participating in the workshop prior to the initial testing.

#### 2.7.1. Recruitment and Baseline Characteristics

Baseline characteristics were compared between groups using multivariate analysis of variance (MANOVA) and Chi-square tests for categorical data (i.e., gender and socio-economic rating). This ensured that any potential difference between the groups post-intervention was not due to pre-existing baseline differences (on the variables that we measured). 

#### 2.7.2. Feasibility and Acceptability of the Exercise Intervention

Feasibility data are presented in the form of attrition and adherence rates, individual item scores, as well as aggregate means and standard deviations. Characteristics of the exercise intervention (intensity, duration, compliance) were compared between groups using MANOVA. Open-ended feedback and recommendations are presented to support the quantitative feasibility measures. 

#### 2.7.3. Preliminary Efficacy of the Intervention

Changes in variables measured both pre- and post-intervention were assessed using a series of 2 (time; pre vs. post) × 2 (group; CEAP vs. control) mixed-model ANOVAs. In instances when a significant time-by-group interaction was observed, follow-up analyses were performed to determine the specific nature of change. Effect sizes were reported as Cohen’s *d* (calculated based on means and standard deviations to indicate pre-to-post change for participants within each condition) or partial eta squared (for associated ANOVAs). Measures that were only completed post-intervention were analyzed using between-group MANOVA.

## 3. Results

### 3.1. Recruitement and Baseline Characteristics

A total of 95 individuals expressed interest in the study and were assessed for eligibility between June 2017 and September 2017, with 45 individuals (men *n* = 15; women *n* = 30) consenting to participate and being randomized to the exercise only (*n* = 21) or the exercise plus CEAP (*n* = 24) (Figure 1). Baseline characteristics of the participants are shown in Table 1. There were no significant differences between the groups (*p* > 0.05) for mean age, weight, BMI, sex, socio-economic background, exercise behavior, exercise motivation, or cognitive restraint of eating; the groups were well-matched on these variables at baseline. With regard to education status, 92% of the participants reported having at least a bachelor’s university degree. 

### 3.2. Assessment of Feasbility of the Exercise Intervention 

A total of eight participants (18%) discontinued the intervention due to medical reasons, pregnancy, or time constraints. One of these participants was in the CEAP group, reflecting a 4% attrition rate for that group, while seven participants assigned to the control condition withdrew from the program, reflecting a 33% attrition rate in control group participants. Excluding dropouts, participants in the two groups did not differ in the average number of exercise sessions attended throughout the intervention (CEAP 21.1 ± 1.2 (88%), control 21.6 ± 1.4 (90%); t (35) = −1.01, *p* = 0.319), with the perceived intensity of exercise performed being similar between groups (session RPE: CEAP 12 ± 0.4, control 13 ± 0.5; t (35) = −1.295, *p* = 0.204). Perceived enjoyment of the intervention program as a whole was also similar between groups (CEAP 4.95 ± 1.11, control 5.33 ± 1.17; t (35) = −0.982, *p* = 0.333). The mixed-model ANOVA for exercise behavior, including both exercise completed as a part of the study and exercise outside of the prescribed sessions (Godin Leisure Time Questionnaire), revealed no significant differences between the groups (F (1, 35) = 0.077, *p* = 0.783, *η*^2^ = 0.002), and no interaction effect between time and group (F (1, 35) = 0.358, *p* = 0.553, *η*^2^ = 0.010). However, there was a significant effect of time from pre- to post-intervention (F (1, 35) = 14.931, *p* < 0.001, *η*^2^ = 0.299), with both the CEAP (t (22) = −3.429, *p* = 0.002) and the control (t (13) = −2.317, *p* = 0.037) groups increasing their exercise levels pre- to post-intervention (excluding dropouts, pre-intervention: CEAP 19.30 ± 14.47, control 20.04 ± 15.06; post-intervention: CEAP 33.65 ± 18.33, control 30.54 ± 14.04). 

#### 3.2.1. CEAP Workshop

Results from the post-workshop evaluation questionnaire assessing the dose received during the CEAP are shown in Table 2. Individual item scores were high, reflecting a positive overall response to the CEAP workshop. 

#### 3.2.2. Use of Behavior Change Techniques

Results for participants’ use of behavior change tools encouraged in the CEAP are shown in Table 2. Mean item scores indicated that there was relatively frequent use of health and wellbeing goals and social support; however, it appeared that participants did not employ self-monitoring or prompts as frequently as may have been expected. For post-intervention measures of action planning, there was no difference between the groups (CEAP 2.26 ± 0.51, control 2.24 ± 0.51; t (34) = 0.111, *p* = 0.912). There was, however, a significant difference between the groups for coping planning, with the CEAP group reporting a higher mean score (CEAP 2.64 ± 0.54, control 2.21 ± 0.67; t (34) = 2.068, *p* = 0.046), and this was accompanied by a moderate-to-large effect size *d* = 0.71. 

#### 3.2.3. Open-Ended Feedback and Recommendations

Twelve out of the 24 participants randomized to the CEAP group completed the seven open-ended feedback questions after the completion of the intervention. Responses to the first two questions assessing the effects of CEAP on compensatory beliefs and behaviors were reviewed positively (e.g., that the information presented was helpful at motivating behavior change). For example, one individual noted “My eating and drinking behavior had definitely improved because of the workshop” (Male, 30), and another participant commented, “I would definitely say that it increased my motivation to do the “right” thing and to not “waste” the time spent on travelling to and from the exercise sessions as well as the exercise itself by not following through on the healthy eating side of things” (Female, 39). Some participants expressed that the information was consistent with their already established knowledge and dietary behaviors, with comments such as “I found the facts and data that was presented was already very common knowledge for me, and was consistent with the ways that I was already trying to live a healthier life” (Female, age 21), and, “I felt that my diet was already fairly regimented. I was already trying to eat as ‘healthy’ as possible and limit ‘junk’ food” (Male, age 39). 

With respect to the behavior change techniques, participants provided several positive comments, including, “Goal-setting is important for me—especially when it involves numbers and graphs and tracking” (Female, age 39). Another participant noted that “The food monitoring diary was useful for helping me see patterns in my intake and why maybe these occurred” (Female, age 33). In relation to social support, one participant commented that “My friend also participated in the study and that was good because we kept each other motivated for exercise and tried to eat more healthily” (Female, age 26). Lastly, for external cues, reminders, and prompts, one participant stated, “I used reminders on my phone for my daily food targets” (Male, age 30). Participants also highlighted important barriers to the implementation of behavior change techniques, providing insight into the optimization of the program in the future. The most frequent responses included believing that dietary change was not a priority or necessity for them as individuals, with comments such as “I definitely thought about my diet; however, as my reasons for exercising were more about de-stressing and cognitive benefits instead of weight, there was not a significant amount of modification” (Female, age 21). In addition, some participants noted that there was a lack of motivation to consistently use the tools; for example, when asked about the self-monitoring food diary, one participant commented “I was definitely focused on this at the beginning, but found as the study went on, I was skipping days or forgetting to write down what I ate. Maybe if this was more formal I would have adhered more” (Male, age 39). 

In response to further recommendations for the workshop, 33% of the participants had no suggestions. The remaining participants noted two predominant recommendations; namely that the workshop be more thorough, as stated by a participant “The seminar should have been more comprehensive” (Female, age 24) and that the program may have benefited from multiple workshops, such as “I think the study was a bit short to significantly change people’s eating habits, particularly with just one workshop. Multiple regular workshops might have been more effective” (Female, age 26). 

### 3.3. Assessment of Preliminary Efficacy of the Intervention 

#### 3.3.1. Compensatory Beliefs and Behaviors

The efficacy of the intervention for altering participants’ CHB scores is shown in Table 3. There was no significant main effect for time (F (1, 34) = 0.006, *p* = 0.940, *η*^2^ < 0.001), or group (F (1, 34) = 1.467, *p* = 0.234, *η*^2^ = 0.041), and no significant interaction effect between time and group (F (1, 34) = 0.035, *p* = 0.853, *η*^2^ = 0.001) for CHB. Likewise, there was no significant main effect of time (F (1, 34) = 1.378, *p* = 0.249, *η*^2^ = 0.039), or group (F (1, 34) = 0.047, *p* = 0.831, *η*^2^ = 0.001), and no significant interaction effect (F (1, 34) = 3.056, *p* = 0.089, *η*^2^ = 0.082) for the initial question of the CEMQ (CEMQ-Q1; “Do you eat more on days that you exercise?”). However, subsequent exploratory paired samples t-tests did reveal a significant decrease between pre- and post-intervention scores for participants in the CEAP group (t (21) = 2.347, *p* = 0.029), but not the control group (t (13) = −0.366, *p* = 0.720), indicating that those in the CEAP group may have reduced their compensatory food intake on their exercise days. Associated effect sizes were negligible for the control group (*d* = 0.08) and small-moderate for the CEAP group (*d* = 0.44). 

#### 3.3.2. Health and Fitness

The effect of the exercise intervention on the physiological health and fitness profile of participants in both the control and CEAP groups, including corresponding effect sizes, is shown in Table 4. There was no significant main effect of group or interaction effect between group and time for any of the physiological health and fitness variables (*p* > 0.05). However, there was a significant main effect for time on waist circumference (F (1, 35) = 28.841, *p* < 0.001, *η*^2^ = 0.452), waist to hip ratio (F (1, 35) = 18.864, *p* < 0.001, *η*^2^ = 0.350), body fat percentage (F (1, 35) = 8.905, *p* = 0.005, *η*^2^ = 0.203), systolic (F (1, 35) = 21.285, *p* < 0.001, *η*^2^ = 0.378) and diastolic (F (1, 35) = 13.271, *p* = 0.001, *η*^2^ = 0.275) blood pressure, and cardiovascular fitness (F (1, 34) = 30.326, *p* < 0.001, *η*^2^ = 0.471). More specifically, waist circumference, waist-to-hip ratio, body fat percentage, and blood pressure values all decreased pre- to post-intervention (irrespective of condition), while cardiovascular fitness increased based on the power output at 80% of HR _max_, with most of these changes supported by small-moderate effect sizes (see Table 4). In contrast, fasting glucose, body mass, and the percentage of lean body mass were not significantly altered pre- to post-intervention (*p* > 0.05) and associated effect sizes were small. 

#### 3.3.3. Snack Intake

There was no significant main effect for time (F (1, 34) = 0.221, *p* = 0.641, *η*^2^ = 0.006) or group (F (1, 34) = 0.355, *p* = 0.555, *η*^2^ = 0.010), and no interaction between time and group (F (1, 34) = 0.009, *p* = 0.927, *η*^2^ < 0.001) for ad libitum energy intake from unhealthy snacks during the laboratory session pre- and post-intervention (pre-intervention: CEAP 462 ± 510, control: 363 ± 544 kJ, post-intervention: CEAP 530 ± 791, control 408 ± 667 kJ). Associated effect sizes from pre- to post-intervention within each group were small (CEAP *d* = 0.10, control *d* = 0.07).

## 4. Discussion

Exercise and food/drinks are commonly paired in a compensatory belief system, and licensing oneself with unhealthy food or drinks around exercise may compromise the achievement of one’s long-term goals during exercise participation or interventions. Our aim in this study was to examine the feasibility and preliminary efficacy of an educational and motivational support program focused on the avoidance of compensatory eating alongside an 8 weeks exercise intervention. Analyses demonstrated that the CEAP is both feasible (i.e., well-received by participants) and may have benefits for reducing compensatory eating around exercise, although any such effects may be small. Self-reported use of some relevant behavior change strategies was lower than expected, which may explain the small effects of the CEAP on compensatory eating behaviors. Therefore, future refinement of the CEAP is warranted prior to implementation in larger-scale trials or community programs. 

Feasibility trials are an important precursor to the implementation of larger (i.e., fully powered) behavior change interventions [40,41,42]. This study demonstrated that the addition of a CEAP to a supervised exercise program is highly feasible from both a participant recruitment and retainment perspective. Recruitment of a university (staff and student) and community cohort occurred without major difficulties (potentially due to the ‘free’ exercise program being offered), and once participants were screened and randomized, attrition rates were favorable. The overall study attrition rate was comparable to other exercise interventions of a similar exercise intensity and duration (16% attrition rate) [43], with a total of 8 participants in the current study failing to complete the intervention (18% attrition rate). When separated by study arm, the attrition rate was considerably higher in the control group compared with the CEAP (33% versus 4%); it is possible that the workshop contact and weekly emails may have aided in motivating participants to follow through with the entire intervention [44]. Regardless, it is noteworthy that the addition of educational and follow-up components (alongside the supervised exercise) within the CEAP did not, in this instance, marginalize participant retention. Compliance to the supervised exercise sessions was also good, with participants completing 89% of the scheduled sessions (after accounting for dropouts). The exercise training was implemented as intended, with a progressive increase in exercise duration and intensity across the intervention period. Lastly, scores on the IMI suggested that cycling three times a week was a relatively enjoyable way of performing exercise within a weekly health routine for previously sedentary populations. Taken together, these findings indicated that the addition of CEAP components (e.g., workshop, weekly reminders, self-monitoring) did not detract from participants’ adherence, attendance, or program enjoyment.

Regarding the primary intervention component (i.e., the CEAP workshop), evaluation measures were collected both immediately after the workshop (to assess the dose received), and at the end of the intervention (to assess the influence and use of behavior change tools). High mean scores for questions assessing the dose received (i.e., “the information on exercise and nutrition was useful in supporting my health objectives”) supported the utility of the CEAP, as did the between-group differences that we observed for the use of coping planning strategies. Relative to the control group, the CEAP group reported higher ratings of coping planning in relation to avoiding compensatory eating at the end of the intervention. Not all behavior change tools incorporated in to the CEAP were highly utilized, however, with relatively low mean scores observed for use of the food-monitoring diaries and external cues, prompts, and reminders. The limited usage of self-monitoring, prompts, cues, and reminders is important to highlight, as previous research has demonstrated that optimal results in behavior change interventions arise when these tools are utilized effectively [13,18,19]. It is possible that a one-time, 1.5 h workshop at the beginning of the intervention may have simply been insufficient to provide participants’ with adequate understanding and motivation regarding the regular and effective use of these tools. This possibility was supported by qualitative feedback, with some participants suggesting that a more comprehensive workshop with multiple follow-up sessions may have helped them better integrate the information and behavior change tools into their exercise program. Accordingly, consistent with some previous diet- and exercise-related interventions, it is possible that additional face-to-face workshops may enhance the effectiveness of these behavior change strategies [45]. Clearly though, repeated face-to-face workshops do place an additional burden on participants, and as such, researchers may also consider less burdensome approaches to encouraging greater use of self-monitoring and prompts (e.g., through mobile applications [46,47]). To strengthen the potential effects of the CEAP intervention on compensatory beliefs and behaviors in the future, researchers may devote greater attention to highlighting the importance of self-monitoring and other practical tools (e.g., cues, reminders, and prompts). 

In addition to examining the feasibility of this CEAP intervention, our secondary aim was to obtain preliminary evidence for (or against) the efficacy of the intervention for improving compensatory beliefs, compensatory behaviors, and downstream health-related outcomes. We observed evidence for potential effects on (self-reported) compensatory eating behavior, with a small-moderate effect size demonstrating reduced compensatory eating behaviors (CEMQ scores) pre- to post-intervention in the CEAP (but not the control) group. Overcompensating with food after exercise is common [4,5] and may contribute to individuals failing to reach their desired health (e.g., weight-loss) goals. It should be noted, however, that the CEMQ item focuses on all food intake and does not discriminate between “healthy” and “unhealthy” options. As a result, the greater food intake reported by control participants was not necessarily reflective of those participants eating more unhealthy food on exercise days. It was partly for this reason that we also utilized the CHB questionnaire [9] (i.e., to examine unhealthy compensatory eating and drinking behaviors). Interestingly, there were no differences in CHB scores between the groups, or from pre- to post-intervention. The reason for the discrepancy between the CEMQ and the CHB may be related, at least in part, to the focus on unhealthy snack foods/drinks specifically rather than food in general. It is also important to note that the mean scores for the CHB scale were relatively low pre-intervention (CEAP 2.62 ± 1.38, control 2.18 ± 0.91) compared with previous studies that have utilized the same instrument (3.19 ± 1.13) [9]. Accordingly, it is possible that there may have been limited opportunities for improvement in the first place (i.e., a floor effect). The majority of participants in this study were also well educated (i.e., 92% of participants held a university degree) and living in relatively high-socioeconomic areas, and this may have contributed to the low CHB scores observed pre-intervention. There is evidence, for example, that individuals from a high socio-economic background possess greater dietary knowledge that supports healthier food choices [48], and analysis of qualitative data indicated that much of the CEAP information relating to exercise and healthy nutrition was already familiar to many participants. Future CEAP workshops with similar populations might, therefore, be adapted to focus more on the practical behavior change tools, rather than educational information. Alternatively, future studies that adopt the same intervention program as this study might be more effective if they are directed toward participants who are less well-educated and from lower socio-economic areas. It is also possible that the non-significant findings for CHB scores may be due, in part, to individuals not being consciously aware that they tend to employ CHBs as a self-regulatory strategy [49], and therefore find it difficult to complete a CHB questionnaire accurately [50]. This is in alignment with research on self-licensing, in which it has been shown that justification cues may influence behavior outside of conscious awareness [11,12]. In addition, individuals might also be prone to socially desirable (biased) responding on CHB questions [51]. With these considerations in mind, the use of implicit CHB measures in future research may provide additional insight into their role in unhealthy compensatory behaviors [52]. 

There were no significant main or interaction effects for ad libitum energy intake from unhealthy snacks, and associated effect sizes were small. Previous work has acknowledged negative associations between CHBs, intention, and action planning to avoid unhealthy snacks [49], as well as a positive relationship between CHBs and unhealthy eating behaviors [53]. In the present study, it was perhaps not surprising that there was no between-group difference in energy intake from unhealthy snacks given that CHBs and action planning did not differ between the groups. In addition, alongside the education and socioeconomic consideration that may have contributed to limited unhealthy food intake, participants were also tested in a fasted state in the morning, and under these conditions it is possible that they may have been unlikely to select chocolate, cookies, and confectionary for “breakfast”. The time of testing may help explain why previous studies have demonstrated contrasting findings regarding compensatory snacking behaviors following exercise [4], with later times of day being associated with higher risk of consuming [54] and craving [55] unhealthy snack food options.

The preliminary efficacy of the intervention for altering commonly assessed health outcomes (e.g., weight loss, blood glucose) was also examined. The regular exercise program (without CEAP) had benefits for waist circumference, body fat percentage, blood pressure, and cardiovascular fitness. These changes confirm the acknowledged benefits of regular exercise on important health markers [56,57]. Interestingly though, and with respect to the efficacy of adding the CEAP to an exercise program, the magnitude of improvements was similar between the two groups. This suggests that the CEAP did not have any additional effects or enhancements on physiological markers of health and fitness in comparison to exercise alone. Considering that there was no significant difference between the groups on both self-reported compensatory eating and levels of physical activity, these similarities in physiological improvements are not surprising.

The findings of this feasibility study must be interpreted in light of design limitations. First, considering that the intervention was advertised as a “healthy lifestyle” intervention and not a “weight-loss” program, it is possible that volunteers did not have the intention of changing dietary behaviors or beliefs, but instead were focused predominantly (or solely) on the exercise component of the intervention. In relation to outcome measures, we did not measure overall dietary composition or overall energy intake at any point during the study. Therefore, future researchers employing interventions such as the CEAP may wish to include measures, such as food records [58,59], to investigate whether dietary composition or overall energy intake change as a result of the intervention. Additionally, the average BMI of the study cohort was within the normal weight range, and on that basis, participants may have been less likely to endorse CHBs relative to overweight or obese individuals [60]. That being the case, it would be worthwhile to examine the efficacy of an integrative intervention such as CEAP in other (e.g., overweight/obese, less educated and/or lower socioeconomic) populations, and specifically among those who strongly endorse weight loss goals. 

## 5. Conclusions

This feasibility study is the first to explore whether CHBs pairing exercise and unhealthy food/drink intake can be altered within an exercise program. Feasibility analyses indicated that there was a low drop-out rate and high mean scores for participant perceptions regarding the primary intervention component (i.e., CEAP workshop). We also observed that the addition of an education and behavior change program (i.e., CEAP) did not result in detrimental effects on participant adherence and enjoyment (relative to exercise-only participants), but did encourage greater use of coping plans throughout the program. This study also provided some evidence for the preliminary efficacy of the CEAP in the form of decreased self-reported compensatory eating behavior; further development is warranted, however, to enhance the scope and magnitude of any effects. With the goal of stimulating more substantial and sustained change in compensatory beliefs and behaviors, we encourage future large-scale trials that more strongly emphasize practical strategies (e.g., self-monitoring, prompts), and that provide more frequent in-program support (e.g., greater face-to-face contact with interventionists, access to mobile applications).

## Figures and Tables

**Table 1:** Baseline characteristics of participants randomized to an 8-week supervised exercise intervention (control) or supervised exercise plus a compensatory eating avoidance program (CEAP) ((mean ± SD)/*n* (%); *p* values).

Baseline Characteristics	Control (*n* = 21)	CEAP (*n* = 24)	*p*
Age (year)	30.90 ± 6.96	29.58 ± 7.25	0.538
Body mass (kg)	68.15 ± 10.53	72.59 ± 13.63	0.233
BMI (kg/m^2^)	23.79 ± 2.55	24.66 ± 3.15	0.323
Female	16 (35.6)	14 (31.1)	0.205
SEIFA IRSAD top quintile (*n* = 44)	14 (70.0)	19 (79.2)	0.484
Leisure time physical activity	21.93 ± 15.28	19.19 ± 14.17	0.536
Exercise motivation	10.42 ± 5.64	9.95 ± 4.10	0.749
Cognitive restraint of eating	2.37 ± 0.50	2.16 ± 0.40	0.136

Note: Body Mass Index (BMI) = indicates body mass index with higher scores reflecting a greater weight-to-height ratio. The Socio-Economic Indexes for Areas: Index of Relative Socio-economic Advantage and Disadvantage (SEIFA IRSAD) top quintile reflects whether an individual’s postal address was within the top quintile of the socio-economic ratings. Leisure time physical activity is measured by Godin’s scale and reflects exercise behavior with higher scores representing a larger amount of physical activity. Exercise motivation, as measured through the Relative Autonomy Index (RAI), reflects greater autonomous (relative to controlled) motivation with higher scores. Cognitive restraint of eating, as measured by the Three Factor Eating Questionnaire (TFEQ), represents an individual’s tendency to restrain their food and drink consumption with higher scores reflecting greater restraint.

**Table 2:** Workshop review ratings from participants randomized to the 8-week supervised exercise plus a compensatory eating avoidance program (CEAP) (mean ± SD).

CEAP Evaluation Items	CEAP Group (*n* = 23)
**Post-Workshop**	
Q1. Overall, the Healthy Lifestyle Workshop was useful in supporting my health objectives.	3.96 ± 0.83
Q2. The information on exercise and nutrition was useful in supporting my health objectives.	3.87 ± 0.82
Q3. The information on compensatory health beliefs and licensing was useful in supporting my health objectives.	4.04 ± 1.07
Q4. The activity on licensing and possible food alternatives was useful in supporting my health objectives.	3.83 ± 0.94
Q5. The activity on goal setting was useful in supporting my health objectives.	4.17 ± 0.72
Q6. The activity on identifying possible barriers and coping plans was useful in supporting my health objectives.	4.26 ± 0.86
Q7. I understood the information that was presented to me during the workshop.	4.91 ± 0.29
Total mean score	4.15 ± 0.65
**Post-Intervention**	
“During the 8-week intervention, to what extent did you…”	
Q1. Stick to the goals you specified in the Healthy Lifestyle Workshop	3.57 ± 0.90
Q2. Use the coping plans formulated in the Healthy Lifestyle Workshop	2.87 ± 1.10
Q3. Enlist social support from others to help you progress toward your health objectives	3.00 ± 1.04
Q4. Use the self-monitoring diary to monitor your food and drink intake around exercise	1.86 ± 1.25
Q5. Use cues, reminders, or prompts to aid your progress toward your health objectives	2.35 ± 1.23
Total mean score	2.73 ± 0.73

**Table 3:** Compensatory Health Beliefs (CHB) and Compensatory Eating Motives Questionnaire (CEMQ-Q1) for supervised exercise training alone (control) or combined with a compensatory eating avoidance program (CEAP) (mean ± SD; Cohen’s *d*).

Compensatory Measures	Control (*n* = 14)			CEAP (*n* = 22)		
	Pre-intervention	Post-intervention	Cohen’s *d*	Pre-intervention	Post-intervention	Cohen’s *d*
CHB	2.18 ± 0.91	2.21 ± 0.83	0.03	2.62 ± 1.38	2.61 ± 0.98	0.01
CEMQ-Q1	2 ± 0.78	2.07 ± 0.92	0.08	2.27 ± 0.77	1.91 ± 0.87	0.44

**Table 4:** Effect of 8 weeks of supervised exercise training alone (control) or combined with a compensatory eating avoidance program (CEAP) on health and fitness profile (mean ± SD; Cohen’s *d; p* values for main effect of time).

Health and Fitness Measures	Control (*n* = 14)			CEAP (*n* = 23)			Main Effect of Time
	Pre-intervention	Post-intervention	Cohen’s *d*	Pre-intervention	Post-intervention	Cohen’s *d*	*p* value
Body mass (kg)	70.23 ± 10.86	69.93 ± 11.11	0.03	72.99 ± 13.79	72.64 ± 13.45	0.03	0.487
BMI	24.30 ± 2.68	24.17 ± 2.49	0.05	24.68 ± 3.22	24.58 ± 3.16	0.03	0.480
Waist circ. (cm)	84.3 ± 8.3	79.4 ± 7.2	0.63	81.8 ± 10.2	79.4 ± 10.4	0.24	<0.001
Waist: Hip ratio	0.82 ± 0.06	0.79 ± 0.06	0.50	0.80 ± 0.06	0.78 ± 0.06	0.33	<0.001
Body fat (%)	35.14 ± 6.40	34.34 ± 6.22	0.13	31.81 ± 9.49	30.81 ± 9.12	0.11	0.005
Lean muscle mass (%)	61.25 ± 6.21	62.12 ± 6.08	0.14	63.42 ± 10.13	64.85 ± 9.21	0.15	0.099
Fasting glucose (mmol/L)	4.6 ± 0.4	4.7 ± 0.6	0.21	4.6 ± 0.3	4.6 ± 0.5	0.17	0.357
Systolic BP (mmHg)	115 ± 9	110 ± 6	0.65	115 ± 12	110 ± 11	0.43	<0.001
Diastolic BP (mmHg)	79 ± 6	73 ± 5	1.09	76 ± 7	74 ± 8	0.27	0.001
Cardiovascular fitness (Power output (W)) at 80% HR_max_	130 ± 31	146 ± 38	0.46	144 ± 35	159 ± 36	0.42	<0.001

Significance *p* > 0.05. Note: BMI = body mass index with higher scores reflecting a greater weight to height ratio. Waist circ. = waist circumference with lower scores reflecting more desirable results. Waist: Hip ratio = waist-to-hip ratio with lower scores reflecting more desirable results. Lean muscle mass % = percentage of lean muscle mass with higher scores reflecting more desirable results. Systolic BP = systolic blood pressure with lower scores reflecting more desirable scores. Diastolic BP = diastolic blood pressure with lower scores reflecting more desirable scores.
